# Outcomes of early catheter ablation for ventricular tachycardia in adult patients with structural heart disease and implantable cardioverter-defibrillator: An updated systematic review and meta-analysis of randomized trials

**DOI:** 10.3389/fcvm.2022.1063147

**Published:** 2022-11-30

**Authors:** Tchavdar Shalganov, Milko Stoyanov, Vassil Traykov

**Affiliations:** ^1^Department of Cardiology, National Heart Hospital, Sofia, Bulgaria; ^2^Department of Cardiology, Acibadem City Clinic Tokuda Hospital, Sofia, Bulgaria

**Keywords:** systematic review, ventricular tachycardia (VT), catheter ablation, all-cause mortality, quality of life

## Abstract

**Aims:**

Catheter ablation (CA) for ventricular tachycardia (VT) can improve outcomes in patients with ischemic cardiomyopathy. Data on patients with non-ischemic cardiomyopathy are scarce. The purpose of this systematic review and meta-analysis is to compare early CA for VT to deferred or no ablation in patients with ischemic or non-ischemic cardiomyopathy.

**Methods and results:**

Studies were selected according to the following PICOS criteria: patients with structural heart disease and an implantable cardioverter-defibrillator (ICD) for VT, regardless of the antiarrhythmic drug treatment; intervention–early CA; comparison–no or deferred CA; outcomes–any appropriate ICD therapy, appropriate ICD shocks, all-cause mortality, VT storm, cardiovascular mortality, cardiovascular hospitalizations, complications, quality of life; published randomized trials with follow-up ≥12 months. Random-effect meta-analysis was performed. Outcomes were assessed using aggregate study-level data and reported as odds ratio (OR) or mean difference with 95% confidence intervals (CIs). Stratification by left ventricular ejection fraction (LVEF) was also done. Eight trials (*n* = 1,076) met the criteria. Early ablation was associated with reduced incidence of ICD therapy (OR 0.53, 95% CI 0.33–0.83, *p* = 0.005), shocks (OR 0.52, 95% CI 0.35–0.77, *p* = 0.001), VT storm (OR 0.58, 95% CI 0.39–0.85, *p* = 0.006), and cardiovascular hospitalizations (OR 0.67, 95% CI 0.49–0.92, *p* = 0.01). All-cause and cardiovascular mortality, complications, and quality of life were not different. Stratification by LVEF showed a reduction of ICD therapy only with higher EF (high EF OR 0.40, 95% CI 0.20–0.80, *p* = 0.01 vs. low EF OR 0.62, 95% CI 0.34–1.12, *p* = 0.11), while ICD shocks (high EF OR 0.54, 95% CI 0.25–1.15, *p* = 0.11 vs. low EF OR 0.50, 95% CI 0.30–0.83, *p* = 0.008) and hospitalizations (high EF OR 0.95, 95% CI 0.58–1.58, *p* = 0.85 vs. low EF OR 0.58, 95% CI 0.40–0.82, *p* = 0.002) were reduced only in patients with lower EF.

**Conclusion:**

Early CA for VT in patients with structural heart disease is associated with reduced incidence of ICD therapy and shocks, VT storm, and hospitalizations. There is no impact on mortality, complications, and quality of life. (The review protocol was registered with INPLASY on June 19, 2022, #202260080).

**Systematic review registration:**

[https://inplasy.com/], identifier [202260080].

## Introduction

Sustained ventricular tachycardia (VT) in patients with structural heart disease is a life-threatening condition posing the risk of syncope and sudden cardiac death, especially with reduced left ventricular ejection fraction (LVEF). Ischemic and non-ischemic cardiomyopathy are the most common conditions associated with VT (1). Most antiarrhythmic drugs are of little value and their use is restricted in patients with LV systolic dysfunction (2). Implantable cardioverter-defibrillators (ICDs) are usually considered first-line treatment in patients with structural heart disease and VT, but this therapy does not prevent VT recurrences and ICD shocks are associated with increased mortality and decreased quality of life (3–7). Several studies and a few meta-analyses in patients with previous myocardial infarction and severe/moderate LV systolic dysfunction have shown that endocardial catheter ablation (CA) can reduce VT recurrences, ICD therapy/shocks, and VT storm (8–11). However, data on patients with non-ischemic cardiomyopathy have shown that ablation more often necessitates an epicardial approach and is less effective, probably due to the more frequent presence of midmyocardial or subepicardial arrhythmogenic substrate (12–15). This additional epicardial approach in patients with diverse etiologies might influence hard clinical outcomes, including all-cause and cardiovascular mortality.

The objective of this systematic review and meta-analysis is to investigate whether early CA with either endocardial or endo-epicardial approach for scar-related monomorphic VT improves outcomes (defined as any appropriate ICD therapy, appropriate ICD shocks, all-cause mortality, VT storm, cardiovascular mortality, cardiovascular hospitalizations, complications, and quality of life) in adult patients with ischemic or non-ischemic cardiomyopathy and ICD regardless of the antiarrhythmic drug treatment, compared to deferred ablation or no ablation.

## Methods

This review was conducted in accordance with the Preferred Reporting Items for Systematic Reviews and Meta-Analyses (PRISMA) guidelines (16) (Supplementary File 1) and is based on a protocol agreed upon by all authors.

### Eligibility criteria

The studies included were selected according to the following PICOS criteria: patients ≥18 years old with structural heart disease and an ICD implanted or planned to be implanted for VT, regardless of the antiarrhythmic drug treatment status; interventional arm with CA performed regardless of the access (endocardial or endo-epicardial) and approach (substrate-guided or electrophysiologically guided ablation); comparison arm with no ablation or deferred ablation; ≥3 of the outcomes reported (see Section “Study selection and outcomes”); published randomized studies with follow-up ≥12 months. The length of follow-up was selected with mortality outcomes in mind.

The exclusion criteria were: studies on patients with hypertrophic cardiomyopathy, myocarditis, Chagas disease, congenital heart diseases, surgical ablation, and stereotactic radioablation.

### Information sources and search strategy

PubMed, Directory of Open Access Journals (DOAJs), and Cochrane Library databases were searched independently by two of the authors (TS and MS) in June 2022. The search was not restricted by language or time period. A search string with the keywords “ablation” AND “ventricular tachycardia” was used. A PubMed search within the title and abstract was done and the results were narrowed using the filter “clinical trial.” DOAJ search was done within the titles, then the search was rerun within the abstracts. The results were narrowed using the filter “medicine.” Cochrane Library was searched within the title, abstract, and keyword with word variations, and the results were filtered using the filter “trials.”

### Study selection and outcomes

Automation tools were not used at any step of the selection process. Duplicate titles were removed. The remaining titles and abstracts were reviewed by all authors independently for appropriateness. All irrelevant publications were removed. The remaining titles were reviewed in full text by the authors independently. All titles that were not rejected/approved unanimously were reviewed again for appropriateness and the final decision was taken after discussion and consensus among all authors. The remaining full-text publications were included in the review and meta-analysis.

The main outcomes analyzed include any appropriate ICD therapy, appropriate ICD shocks, all-cause mortality, and VT storm. Additional outcomes include cardiovascular mortality, cardiovascular hospitalizations, and serious adverse events related to the assigned treatment. Quality of life (physical and mental components) at the last visit was added to the additional outcomes with the amendment of the review protocol.

### Data extraction and risk of bias assessment

Data on the outcomes and other variables were extracted independently by two of the authors (TS and VT) from the full texts and any supplementary files available into standardized Excel tables, and verified and approved by the third author (MS). Detailed data on quality of life were requested by e-mail from the first authors of three of the trials.

Other data extracted for the systematic review included: first author, year of publication, overall sample size, sample size of the experimental and comparison arms, comparator, inclusion criteria, ablation procedure design, access to the heart, use of navigation system, procedural end-point, primary outcome, secondary outcomes, age, sex, New York Heart Association class, LVEF, hypertension, diabetes mellitus, use of beta-blockers, amiodarone, angiotensin-converting enzyme inhibitors (ACEIs) or angiotensin receptor blockers (ARBs), and mineralocorticoid receptor antagonists, percentage of cardiac resynchronization therapy system with defibrillator (CRT-D) implants, percentage of ICDs implanted before the ablation, length of follow-up, cross-over to the ablation arm, and serious adverse events related to the study procedures.

Risk of bias assessment was done using the revised Cochrane Risk of Bias 2 tool (RoB 2) for randomized trials in categories: selection (random sequence generation and allocation concealment), performance (blinding of participants and personnel), detection (measurement of the outcome), attrition (missing outcome data), reporting (selection of the reported result), and others. The assessment was done by all authors independently. Disagreements were resolved by discussion and consensus.

### Effect measures and synthesis methods

Outcomes were assessed using aggregate study-level data. Dichotomous outcomes are reported as odds ratio (OR) with 95% confidence intervals (CIs). The difference in the quality of-life scores between the experimental and control arms at the last visit is reported as a mean difference (MD) with 95% CI. A *p*-value < 0.05 for the overall effect was considered significant.

Studies were deemed eligible for each synthesis if the specific outcome was reported.

Data for dichotomous outcomes and quality-of-life scores were extracted directly from the study report and/or the supplementary files.

The results of the syntheses are presented as forest plots with studies arranged in descending order by publication date. Plots include the number of events (or the mean difference), total number of patients in each arm of each study, the overall number of events and patients, and the weight of the study. Statistical tests for heterogeneity and for overall effect are also shown in the forest plots. The main characteristics of the studies are shown in Tables 1, 2 arranged by publication date.

**TABLE 1:** Main characteristics of the studies.

Study	SMASH-VT	VTACH	VANISH	SMS	BERLIN VT	PARTITA	SURVIVE-VT	PAUSE-SCD
Author	Reddy	Kuck	Sapp	Kuck	Willems	Della Bella	Arenal	Tung
Year	2007	2010	2016	2017	2020	2022	2022	2022
Sample size (n)	128	107	259	111	159	47	144	121
Sample size experimental/ comparator (n)	64/64	52/55	132/127	54/57	76/83	23/24	71/73	60/61
Experimental arm	ICD + pre- or post-ICD ablation	Ablation followed by ICD implantation	ICD + ablation	Ablation followed by ICD implantation	Ablation followed by ICD implantation	ICD + ablation	ICD + ablation	ICD + pre- or post-ICD ablation
Comparator arm	ICD alone, no ablation	ICD alone, no ablation	ICD + AAD escalation, no ablation	ICD alone, no ablation	ICD implantation + deferred ablation (after 3 ICD shocks)	ICD alone, no ablation	ICD + AAD, no ablation	ICD alone, no ablation
Inclusion criteria	Age >18 years, history of MI (documented by ECG or cardiac imaging) and a planned or a recent (within 6 month) implantation of an ICD for VF, hemodynamically unstable VT, or syncope with inducible VT during invasive EPS; patients with an ICD for primary prevention and with subsequent appropriate ICD therapy for a single event	Age 18–80 years, indication for a secondary prevention ICD after documented stable clinical VT without reversible cause, CAD, previous MI, and reduced LVEF ≤50%	Previous MI, an ICD implanted, and an episode of VT during treatment with amiodarone or another class I or class III AAD within the previous 6 months	Age 18–80 years, CAD, LVEF ≤40% and clinically unstable spontaneous VT, or cardiac arrest, or syncope with unstable VT inducible at the baseline EPS	Previous MI (>4 weeks), LVEF 30–50%), life-threatening ventricular arrhythmias necessitating ICD implantation, including documented sustained VT	ICM or NICM and primary or secondary prevention indication for ICD, after having received 1st appropriate shock for VT after the observational phase	Previous MI (>6 weeks), optimal medical treatment (if ventricular dysfunction), and an episode of very symptomatic VT defined as: (1) sustained VT treated using ICD shock (<6 months); and (2) sustained VTs with syncope, even if terminated with ATP; monomorphic VT necessitating ICD; not receiving AAD (amio + bb or sotalol + bb or amio)	Age >18 years, ICM, DCM, ARVC, EF <50% and an indication for an ICD for secondary prevention of monomorphic VT or criteria for primary prevention ICD with inducible monomorphic VT during EPS
Procedure design	Prophylactic substrate ablation in sinus rhythm–linear lesions at good pace-mapping sites, ablation of late/fractionated potentials, encirclement of small infarcts	Prophylactic ablation in stable VT; substrate modification in non-inducible or unstable VT by pace-mapping based linear lesions	Activation mapping and ablation of all inducible VTs; if not tolerated–substrate ablation guided by pace-mapping and late potentials.	Prophylactic ablation of all inducible VTs; substrate modification.	Preventive ablation of late potentials	Late potential ablation in sinus rhythm followed (if necessary) by activation mapping and ablation in VT	Substrate ablation in sinus rhythm–late potentials, conducting channels, pace-mapping; AAD not allowed in the ablation arm	Substrate ablation in sinus rhythm–late/abnormal potentials within the scar, pace-mapping; activation mapping in stable VT
Access	Endocardial	Endocardial	Endocardial	Endocardial	NR	Endocardial/endo-epicardial	Endocardial	Endocardial/ endo-epicardial
EAM	CARTO	CARTO/EnSite	Yes (NS)	CARTO/EnSite	Yes (NS)	CARTO/EnSite	CARTO/EnSite	EnSite
Procedure end-point	NR (non-inducibility?)	Non-inducibility or abolition of all channels within the scar	Termination and non-inducibility of VT	Non-inducibility	Abolition of all late potentials and non-inducibility	Abolition of late potentials and non-inducibility	Elimination of all arrhythmogenic substrate (post-ablation induction of VT avoided)	Non-inducibility of targeted VT and elimination of abnormal EGMs; complete non-inducibility not mandatory
Primary outcome	Survival free from any appropriate ICD therapy (shocks or ATP)	Time from ICD implantation to recurrence of any sustained VT or VF	Composite of death at any time after randomization or VT storm or appropriate ICD shock after a 30-day treatment period	Time to first recurrence of VT/VF	Composite of all-cause death and unplanned hospitalization for either symptomatic ventricular tachyarrhythmia or worsening HF	Composite of death from any cause or worsening HF that led to hospitalization	Composite of CV death, appropriate ICD shock, unplanned hospitalization for worsening HF, or severe treatment-related complications	Composite of VT recurrence (any appropriate ICD therapy or documented SMVT), CV hospitalization (for HF, complications or arrhythmia), or death
Secondary outcomes	Freedom from any appropriate ICD shock, death, and ICD storm	Survival free from death, syncope, hospital admission for a cardiac reason, and VT storm	Each of the primary outcome components and adverse effects	Appropriate ICD therapies, QoL, hospital readmissions because of a cardiac indication, and severe clinical events (death, number of syncope and of electrical storm episodes)	Sustained VT or VF, appropriate ICD therapy, inappropriate ICD therapy, death from any cause, death from cardiac causes, unplanned hospitalization for any cause, unplanned hospitalization for cardiac reasons, change in QoL, short-term success and complications of ablation, all serious adverse events	Death from cardiac causes, recurrences of sustained VT or VF, appropriate ICD therapy, electrical storm	Each of the primary outcome components as well as sustained VT or VF, appropriate and inappropriate ICD therapies, death from any cause, unplanned hospitalization for ventricular arrhythmias and cardiac events, change in LVEF, and QoL	Each of the primary outcome components

AAD, antiarrhythmic drug; amio, amiodarone; ARVC, arrhythmogenic right ventricular cardiomyopathy; ATP, antitachycardia pacing; bb, beta-blocker; CAD, coronary artery disease; CV, cardiovascular; DCM, dilated cardiomyopathy; EAM, electroanatomic mapping; EF, ejection fraction; EGM, electrogram; EPS, electrophysiologic study; HF, heart failure; ICD, implantable cardioverter-defibrillator; ICM, ischemic cardiomyopathy; MI, myocardial infarction; NA, not available/applicable; NICM, non-ischemic cardiomyopathy; NR, not reported; NS, not specified; QoL, quality of life; SMVT, sustained monomorphic VT; VT, ventricular tachycardia; VF, ventricular fibrillation.

**TABLE 2:** Main characteristics of the patient population of the studies analyzed.

Study	SMASH-VT	VTACH	VANISH	SMS	BERLIN VT	PARTITA	SURVIVE-VT	PAUSE-SCD
Author	Reddy	Kuck	Sapp	Kuck	Willems	Della Bella	Arenal	Tung
Year	2007	2010	2016	2017	2020	2022	2022	2022
Sample size (n)	128	107	259	111	159	47	144	121
Intervention/control sample size (n)	64/64	52/55	132/127	54/57	76/83	23/24	71/73	60/61
Age (years)	67 ± 9/66 ± 10	67.7 ± 8.3/64.4 ± 8.2	70.3 ± 7.3/67.0 ± 8.6	68.4 ± 7.7/65.9 ± 8.4	66 ± 10/66 ± 9	71.2 ± 8.1/65.6 ± 9.6	70 (63–75)/71 (64–76)	51 (45.5–65)/57 (47–63)
Males (%)	92/81	96/91	92.9/93.2	87/81	88.2/86.7	83/88	98.6/93.2	73.3/88.5 (*p* = 0.03)
NYHA class (%) (ablation/control	84/77% in I-II; 16/23% in III-IV	NR (class IV excluded)	77.3/75.5% in I-II; 22.7/24.4% in III	NR (class IV excluded)	77.6/78.3% in I-II; 22.4/21.7% in III	87/81% in I-II; 13/19% in III	91.4/93.2% in I-II; 8.6/6.8% in III	76.7/83.6% in I-II; 20.3/13.1% in III; 3.3/3.3% in IV
LVEF (%)	30.7 ± 9.5/32.9 ± 8.5	34 ± 9.6/34.1 ± 8.8	31.2 ± 10.7/31.1 ± 10.4	32 ± 6.9/30.4 ± 7.3	41 ± 6/41 ± 6	31.9 ± 9/32.4 ± 8.3	35 (26–41)/33 (25–40)	41 (31–60)/40 (30–48)
HTN (%)	73/67	NR	69.3/69.7	NR	81.6/79.5	81/68	78.9/64.4	31.7/34.4
DM (%)	38/50	NR	31.5/28.0	NR	30.3/26.5	19/41	29.6/20.5	13.3/24.6
b-blocker (%)	94/98	75/75	93.9/96.1	91/91	76.3/71.1	100/100	97.2/86.1	78.3/86.9
ACEI/ARB (%)	92/92	NR	87.9/87.4	90/100	61.8/71.1	87/92	98.6/90.3	43.3/50.8
MRA (%)	NR	NR	NR	NR	23.7/25.3	NR	55.7/60.9	NR
Amiodarone (%)	0/0 (exclusion criterion)	35/35	64.4/66.1	30/35	40.8/26.5	5/21	0/87.1	33.3/37.7
CRT-D (%)	0/0	0/0	22/17.3	9.3/10.5	11/3.6	30/33	15.5/18.1	6.7/6.6
ICD implanted before the ablation	87%	7.7%	100%	11%	0%	100%	95.8%	NR
Follow-up (months)	22.5 ± 5.5	22.5 ± 9	27.9 ± 17.1	27.6 ± 13.2	13.2 ± 9.5	24.2 (8.5–24.4)	23.8 (16.6–24)/23.3 (9.4–23.9)	31.3 (20.1–40)
Cross-over to ablation (n)	NR	12	11	1	8	1	18	NR
Severe complications related to the study procedures(n)	3/0	6/9	20/39	13/9	14/8	0/0	7/21	5/0

Cells with two numbers present intervention/control arms. The central tendency is presented as mean ± SD or median (IQR 25–75%). ACEI, Angiotensin-converting enzyme inhibitor; ARB, angiotensin receptor blocker; CRT-D, cardiac resynchronization therapy system with defibrillator; DM, diabetes mellitus; HTN, hypertension; ICD, implantable cardioverter-defibrillator; LVEF, left ventricular ejection fraction; MI, myocardial infarction; MRA, mineralocorticoid receptor antagonist; NA, not available/applicable; NYHA, New York Heart Association; NR, not reported.

We conducted a random-effect meta-analysis using the inverse-variance DerSimonian–Laird model estimator. An outcome less frequently present in the experimental intervention arm has an OR < 1. A positive value of MD favors the experimental arm. Heterogeneity between studies was assessed with the Cochrane *Q* statistic and the *I*^2^ statistic. Values of the *I*^2^ statistic ≤50% were considered to show low/moderate heterogeneity, while those >50% were accepted as designating substantial/considerable heterogeneity. Review Manager 5.4 (17) was used for writing the review and performing the statistics.

Sensitivity analysis was done using the leave-one-out approach by excluding consecutively one study at a time.

Funnel plots for publication bias were constructed. Funnel plot asymmetry was assessed by the R package metafor for jamovi 2.3 (18) using rank correlation and regression tests. *p*-value < 0.05 was considered significant.

### Certainty assessment

Certainty assessment was done using the Grading of Recommendations Assessment, Development, and Evaluation (GRADE) working group tool (19). All authors had access to the tool and worked independently. Outcomes were assessed in the following domains: risk of bias, inconsistency, indirectness, and imprecision. The results are presented in a summary-of-findings table including absolute and relative measures of effect with CIs and the certainty of evidence.

## Results

### Search results and study selection

**FIGURE 1:**
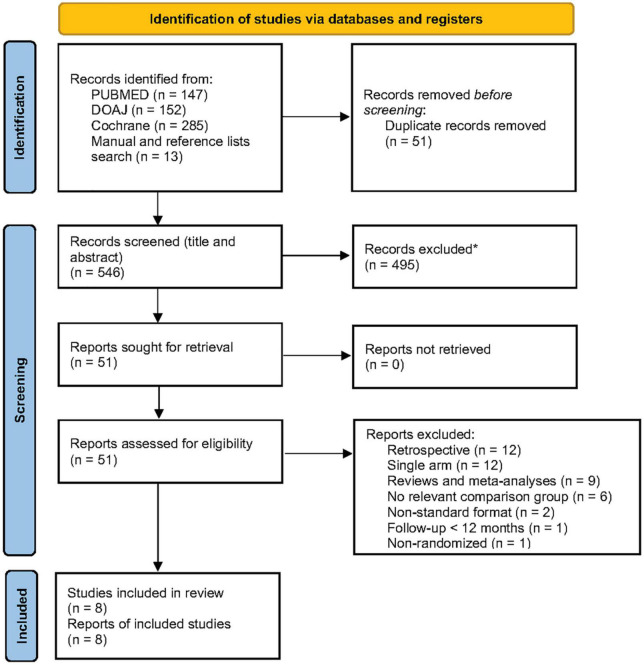
Preferred Reporting Items for Systematic Reviews and Meta-Analyses (PRISMA) flowchart for study identification and screening. *Automation tools were not used. From: Page et al. (16). For more information, visit: http://www.prisma-statement.org/.

Overall, 597 records were identified. After the removal of duplicates and irrelevant studies, 51 reports were assessed for eligibility. Forty-three reports were excluded with reasons, and the remaining eight studies were included in the review (Figure 1). The search was repeated using the same criteria on August 7, 2022 without providing any new studies fulfilling the eligibility criteria.

Several studies that were deemed unsuitable for this review deserve to be mentioned. The short follow-up of only 6 months was the single reason to exclude the small randomized feasibility CALYPSO pilot trial (20). With the two mortality outcomes in mind, we had decided beforehand to include studies with longer follow-ups thus allowing for more events. Another large prospective study on 206 patients (non-ischemic cardiomyopathy in 43%) by Acosta et al. (21) fulfilled almost all inclusion criteria except for its non-randomized design and only a few outcomes were reported. Two Japanese studies were also excluded (22, 23). Both together had almost 190 patients with different etiologies of structural heart disease, but were retrospective and single-arm, and reported only a limited number of outcomes. Finally, a recent study by Yadav with 72 patients had to be removed from further review as well due to its non-randomized nature and only a few outcomes reported (24).

### Study characteristics

Eight randomized clinical trials satisfying the criteria were included in this review and meta-analysis–SMASH-VT (25), VTACH (26), VANISH (27), SMS (28), BERLIN VT (29), PARTITA (30), SURVIVE-VT (31), and PAUSE-SCD (32). These trials enrolled overall 1,076 patients–532 in the early ablation arm and 544 in the control arm. The main characteristics of the studies are shown in Tables 1, 2.

The design of the ablation procedure in all trials included some type of arrhythmogenic substrate modification. Two of the most recent studies (30, 32) allowed epicardial or endo-epicardial access. The procedural end-point was predominantly non-inducibility and abolition of the abnormal signals within the scar. PAUSE-SCD did not mandate complete non-inducibility, while in SURVIVE-VT post-ablation VT induction was even avoided. The etiology of structural heart disease was mainly ischemic. Two recent trials (30, 32) included patients with non-ischemic cardiomyopathy (pooled *n* = 46) and arrhythmogenic cardiomyopathy (*n* = 42), accounting for 8.2% of the pooled patient population. The majority of the studies (25–28, 30, 31) had a mean/median follow-up of approximately 2 years, with the shortest follow-up being 13 months (29), while the longest one reached 31 months (32).

The use of beta-blockers was around 75% in three studies (26, 29, 32) and >90% in the other five studies. ACEIs/ARBs were used in at least 87% of the patients in five studies (25, 27, 28, 30, 31). Two studies had lower use of ACEI/ARB (29, 32), while one did not report data on these drugs (26). Only one study reported the use of mineral-corticoid receptor antagonists in one-quarter of the patients (29), another one reported combined data on renin–angiotensin–aldosterone system inhibitors (31), while in the remaining six trials data were not reported. The use of amiodarone was quite variable across the trials and was an exclusion criterion in SMASH-VT and in the ablation arm of SURVIVE-VT. The overwhelming majority of the patients were men at a mean age of 65–70 years, hypertension was present in 70–80%, and diabetes mellitus was present in around 30%. Two trials did not enroll patients with NYHA class IV heart failure and did not report the NYHA class of the enrolled patients (26, 28), while in the other six trials 75–92% of the patients were in NYHA class I or II. Only two studies enrolled patients in NYHA class IV (25, 32), but only one of them reported specifically that these were 3.3% of the study population (32). In most trials, the mean LVEF was 30–35% except for BERLIN VT and PAUSE-SCD where it was 40%. Relatively more women were enrolled in PAUSE-SCD, and overall, the patients in this trial were younger, with less severe LV dysfunction, and had less hypertension and diabetes. Cross-over to ablation was reported in six studies for overall 51 patients.

Implantable cardioverter-defibrillator was implanted before the ablation in 87–100% of the patients in the ablation arm in four trials (25, 27, 30, 31), and in 8–11% in two other trials (26, 28). All patients in BERLIN VT had the ICD implanted after the ablation. In PAUSE-SCD, the “ablation was done a median 2 days before ICD implantation (IQR, 5 days before to 14 days after)” (32). CRT-D was implanted in 7–31% of the patients in six trials (27–32) and in none of the patients in the other two trials.

Quality of life was assessed in 198 patients in three studies (28, 29, 31) using the Medical Outcomes Short Form-36 general health survey (SF-36). Although SF-36 quality of life data was collected and reported also in VTACH and VANISH (33), the data were presented in a way precluding comparison and meta-analysis.

### Risk of bias

**FIGURE 2:**
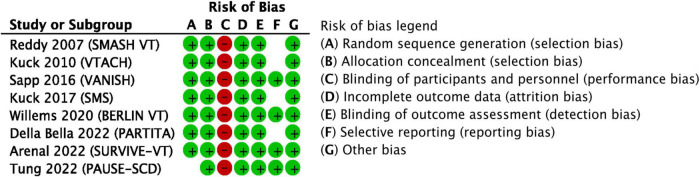
Results of the overall risk of bias assessment. Red circle–high risk, green circle–low risk, empty space–unclear risk.

The risk of bias for all domains in all studies is shown in Figure 2. One article (32) reported a switch to central randomization, while the study was ongoing and after having enrolled approximately 20% of all patients. This study showed significant male prevalence in the control arm and no differences in the other baseline characteristics. Although this difference could be due to a play of chance, we preferred to be conservative and to label the random sequence generation risk of bias as unclear. The risk of performance bias was unavoidably high in all studies given the invasive nature of the intervention and the lack of a sham control procedure. Three studies (25, 28, 32) did not report whether the outcome assessors were blinded to the intervention; however, the detection bias was deemed low. The reporting bias was deemed unclear in studies where we were not able to find a protocol published ahead of the main results article or where the protocol was significantly amended during the course of the study (25, 26, 28, 30). In all other domains, the risk of bias was deemed low. We have to note that specifically for the secondary outcome “quality of life” the risk of attrition bias and the risk of bias in the outcome measurement were high in all studies reporting this outcome (28, 29, 31).

### Outcomes–Results of syntheses

All studies reported appropriate ICD shocks, all-cause mortality, cardiovascular mortality, and adverse events related to the study procedures (no complications were reported in PARTITA and individual OR for this study was not estimable; however, this did not impact the pooled OR). Cardiovascular hospitalizations were reported by seven studies, any appropriate ICD therapy and VT storm were reported by six studies, and changes in the quality of life–by three studies.

**FIGURE 3:**
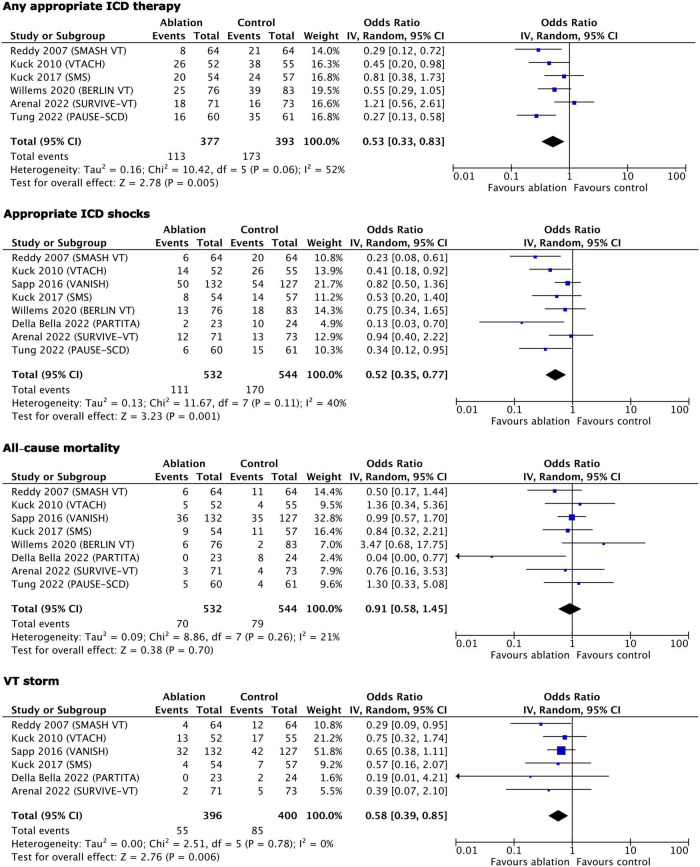
Main outcomes–results of quantitative synthesis.

**FIGURE 4:**
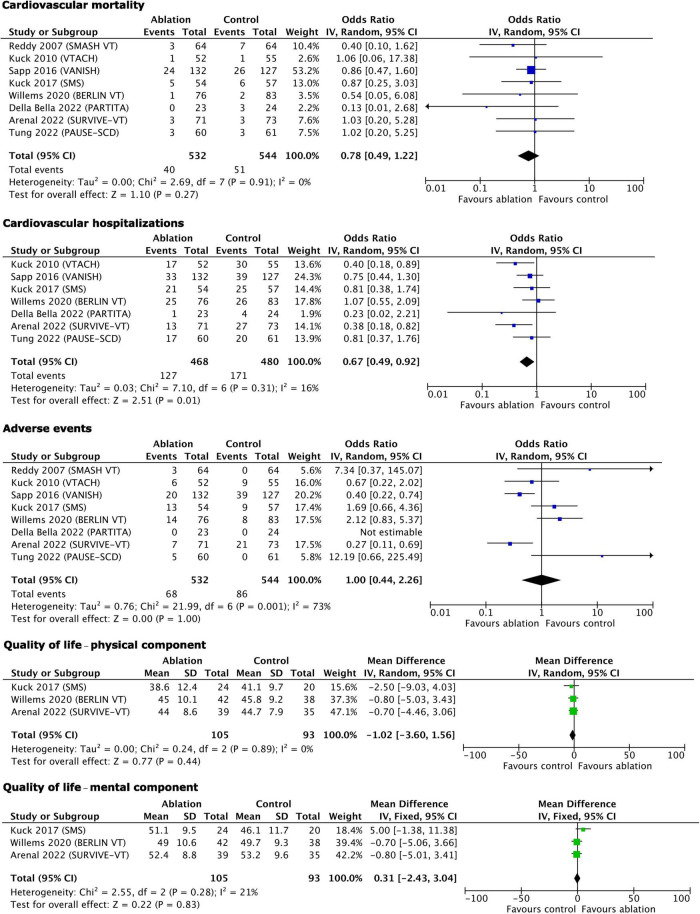
Secondary outcomes–results of quantitative synthesis.

The results of the statistical syntheses of the primary and secondary outcomes are presented as forest plots in Figures 3, 4.

Within the primary outcomes, significant benefits from early ablation were found for any appropriate ICD therapy (OR 0.53, 95% CI 0.33–0.83, *p* = 0.005), appropriate ICD shocks (OR 0.52, 95% CI 0.35–0.77, *p* = 0.001), and VT storm (OR 0.58, 95% CI 0.39–0.85, *p* = 0.006). There was no effect of early ablation on all-cause mortality (OR 0.91, 95% CI 0.58–1.45, *p* = 0.7).

Among the secondary outcomes, only cardiovascular hospitalizations benefited from early ablation (OR 0.67, 95% CI 0.49–0.92, *p* = 0.01). Cardiovascular mortality (OR 0.78, 95% CI 0.49–1.22, *p* = 0.27), complications/serious adverse events (OR 1.00, 95% CI 0.44–2.26, *p* = 1.00), physical component of the quality of life (MD −1.02, 95% CI −3.60 to +1.56, *p* = 0.44), and mental component of quality of life (MD 0.31, 95% CI −2.43 to +3.04, *p* = 0.83) were not influenced by early ablation. With regard to the adverse events, it is worth emphasizing that only study procedure-related events were counted as follows: (1) Ablation-related complications and ICD implantation-related complications (in studies where ICD was implanted peri-ablation) in the early ablation group; (2) ICD implantation-related complications in the control group in trials where ICD had to be implanted peri-randomization; (3) ablation-related adverse events in the control group with deferred ablation; and (4) antiarrhythmic drug treatment-related events in the control group in trials comparing ablation to antiarrhythmic drug treatment.

**FIGURE 5:**
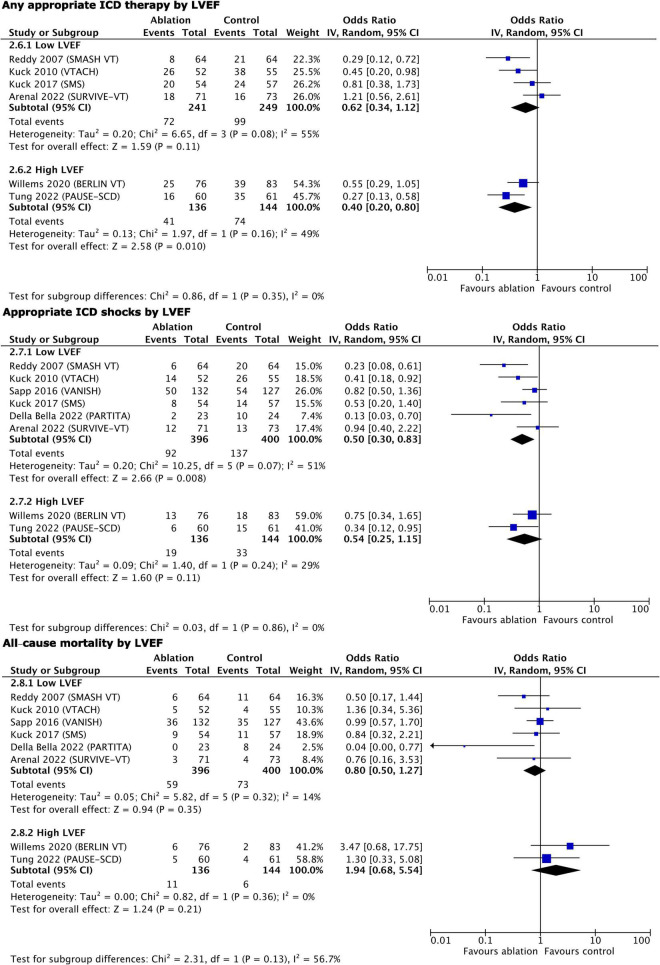
Main outcomes, stratified by LVEF–results of quantitative synthesis.

**FIGURE 6:**
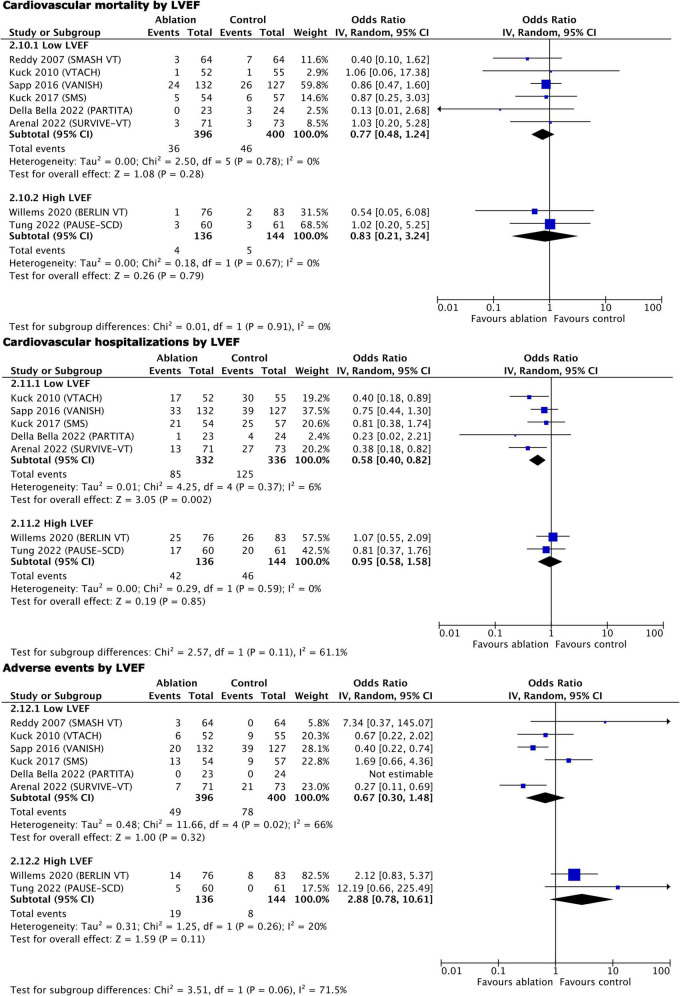
Secondary outcomes, stratified by LVEF–results of quantitative synthesis.

Two of the studies reported a higher mean LVEF of 40% (29, 32); thus, we performed a subgroup analysis of all primary and secondary outcomes except for VT storm and quality of life, stratified by LVEF (Figures 5, 6). Early ablation favored reduction of any appropriate ICD therapy only with higher LVEF (high LVEF OR 0.40, 95% CI 0.20–0.80, *p* = 0.01 vs. low LVEF OR 0.62, 95% CI 0.34–1.12, *p* = 0.11; *p* = 0.35 for subgroup differences). On the contrary, only trials with lower LVEF showed a reduction of appropriate ICD shocks (high LVEF OR 0.54, 95% CI 0.25–1.15, *p* = 0.11 vs. low LVEF OR 0.50, 95% CI 0.30–0.83, *p* = 0.008; *p* = 0.86 for subgroup differences), as well as of cardiovascular hospitalizations (high LVEF OR 0.95, 95% CI 0.58–1.58, *p* = 0.85 vs. low LVEF OR 0.58, 95% CI 0.40–0.82, *p* = 0.002; *p* = 0.11 for subgroup differences). All-cause mortality remained unaffected by LVEF stratification (high LVEF OR 1.94, 95% CI 0.68–5.54, *p* = 0.21 vs. low LVEF OR 0.80, 95% CI 0.50–1.27, *p* = 0.35; *p* = 0.13 for subgroup differences), as did cardiovascular mortality (high LVEF OR 0.83, 95% CI 0.21–3.24, *p* = 0.79 vs. low LVEF OR 0.77, 95% CI 0.48–1.24, *p* = 0.28; *p* = 0.91 for subgroup differences) and complications (high LVEF OR 2.88, 95% CI 0.78–10.61, *p* = 0.11 vs. low LVEF OR 0.67, 95% CI 0.30–1.48, *p* = 0.32; *p* = 0.06 for subgroup differences).

### Heterogeneity and sensitivity analysis

The *Q* and *I*^2^ statistics showed only low to moderate heterogeneity for all primary outcomes, except for any appropriate ICD therapy where the *Q* statistic showed a strong tendency to significance (*p* = 0.06), while the *I*^2^ had a value of 52% pointing formally to substantial heterogeneity (Figure 3). Similarly, the *Q* and *I*^2^-tests for the secondary outcomes showed low heterogeneity, except for the complications related to the assigned treatment where it was substantial/considerable (Figure 4).

Leave-one-out sensitivity analysis was done for all primary and secondary outcomes. The removal of one study at a time resulted in no significant change in OR, MD, and CI for neither of the outcomes, demonstrating the robustness of the main analyses. The results after the removal of the study with the largest weight are shown in Table 3. In most cases, this was VANISH, being the study with the largest population enrolled and consequently with the highest number of events during the follow-up. BERLIN VT had the largest weight for the outcome “any appropriate ICD therapy,” while SURVIVE-VT had the largest weight for the two quality-of-life outcomes.

**TABLE 3:** Sensitivity analysis–Leave-one-out approach.

Outcome (number of studies in the main/sensitivity analysis)	Main analysis	Sensitivity analysis
Any appropriate ICD therapy (6/5)	OR 0.53 (95% CI 0.33–0.83), *p* = 0.005	OR 0.52 (95% CI 0.29–0.92), *p* = 0.02
ICD shocks (8/7)	0.52 (95% CI 0.35–0.77), *p* = 0.001	0.46 (95% CI 0.30–0.71), *p* = 0.0004
All-cause mortality (8/7)	OR 0.91 (95% CI 0.58–1.45), *p* = 0.70	OR 0.88 (95% CI 0.47–1.66), *p* = 0.70
VT storm (6/5)	OR 0.58 (95% CI 0.39–0.85), *p* = 0.006	OR 0.51 (95% CI 0.29–0.90), *p* = 0.02
Cardiovascular mortality (8/7)	OR 0.78 (95% CI 0.49–1.22), *p* = 0.27	OR 0.69 (95% CI 0.36–1.33), *p* = 0.27
Cardiovascular hospitalizations (7/6)	OR 0.67 (95% CI 0.49–0.92), *p* = 0.01	OR 0.64 (95% CI 0.43–0.95), *p* = 0.03
Complications (8/7)	OR 1.00 (95% CI 0.44–2.27), *p* = 1.00	OR 1.28 (95% CI 0.50–3.32), *p* = 0.61
QoL physical component (3/2)	MD −1.02 (95% CI −3.60 to +1.56), *p* = 0.44	MD −1.30 (95% CI −4.85 to +2.25), *p* = 0.47
QoL mental component (3/2)	MD 0.31 (95% CI −2.43 to +3.04), *p* = 0.83	MD 1.11 (95% CI −2.49 to +4.72), *p* = 0.54

CI, confidence interval; ICD, implantable cardioverter-defibrillator; MD, mean difference; OR, odds ratio; QoL, quality of life; VT, ventricular tachycardia.

### Reporting biases and certainty of evidence

The rank correlation and regression tests showed potential funnel plot asymmetry only for the outcome “Appropriate ICD shocks” (*p* = 0.031 and *p* = 0.008, respectively, for the two tests). Funnel plots for all main and secondary outcomes are shown in Supplementary Figure 1.

The quality-of-life outcomes were obviously biased due to significant attrition of follow-up data–only 198 out of 414 patients had an assessment at the last visit. Besides, quality of life is a self-reported outcome (i.e., the participants are outcome assessors) and, given the fact that all studies were open-label, it is possible that the participants were biased in some way by the assigned intervention.

The summary of the findings according to the certainty of evidence (GRADE) is shown in Table 4.

**TABLE 4:** Summary of findings.

Outcomes effect measure follow-up	Number of participants (studies)	Certainty of the evidence (GRADE)	Relative effect (95% CI)	Anticipated absolute effects	
				Risk with deferred ablation or no ablation	Risk difference with early ablation*
Any appropriate ICD therapy–assessed with: odds ratio Follow-up: mean 23.4 months	770 (6 RCTs)	⊕⊕⊕○ Moderate^a^	OR 0.53 (0.33 to 0.83)	440 per 1,000	146 fewer per 1,000 (234 fewer to 45 fewer)
Appropriate ICD shocks–assessed with: odds ratio Follow-up: mean 24.2 months	1,076 (8 RCTs)	⊕⊕⊕○ Moderate^a^	OR 0.52 (0.35 to 0.77)	313 per 1,000	121 fewer per 1,000 (175 fewer to 53 fewer)
All-cause mortality–assessed with: odds ratio Follow-up: mean 24.2 months	1,076 (8 RCTs)	⊕⊕⊕○ Moderate^b^	OR 0.91 (0.58 to 1.45)	145 per 1,000	11 fewer per 1,000 (56 fewer to 52 more)
VT storm–assessed with: odds ratio Follow-up: mean 24.7 months	796 (6 RCTs)	⊕⊕⊕⊕ High	OR 0.58 (0.39 to 0.85)	213 per 1,000	77 fewer per 1,000 (117 fewer to 26 fewer)
CV mortality–assessed with: odds ratio Follow-up: mean 24.2 months	1,076 (8 RCTs)	⊕⊕⊕○ Moderate^b^	OR 0.78 (0.49 to 1.22)	94 per 1,000	19 fewer per 1,000 (46 fewer to 18 more)
CV hospitalizations–assessed with: odds ratio Follow-up: mean 24.3 months	948 (7 RCTs)	⊕⊕○○ Low^b^	OR 0.67 (0.49 to 0.92)	356 per 1,000	86 fewer per 1,000 (143 fewer to 19 fewer)
Complications–assessed with: odds ratio Follow-up: mean 24.2 months	1,076 (8 RCTs)	⊕○○○ Very low^c^	OR 1.00 (0.44 to 2.26)	158 per 1,000	0 fewer per 1,000 (80 fewer to 149 more)
QoL physical component–assessed with: mean difference Scale from: 0 to 100 Follow-up: mean 20 months	198 (3 RCTs)	⊕○○○ Very low^b,d,e^	–	The mean QoL physical component was 0	MD 1.02 lower (3.6 lower to 1.56 higher)
QoL mental component–assessed with: mean difference Scale from: 0 to 100 Follow-up: mean 20 months	198 (3 RCTs)	⊕○○○ Very low^b,d,e^	–	The mean QoL mental component was 0	MD 0.31 higher (2.43 lower to 3.04 higher)

GRADE Working Group grades of evidence High certainty: we are very confident that the true effect lies close to that of the estimate of the effect. Moderate certainty: we are moderately confident in the effect estimate: the true effect is likely to be close to the estimate of the effect, but there is a possibility that it is substantially different. Low certainty: our confidence in the effect estimate is limited: the true effect may be substantially different from the estimate of the effect. Very low certainty: we have very little confidence in the effect estimate: the true effect is likely to be substantially different from the estimate of effect. *The risk in the intervention group (and its 95% confidence interval) is based on the assumed risk in the comparison group and the relative effect of the intervention (and its 95% CI). CI, confidence interval; CV, cardiovascular; MD, mean difference; OR, odds ratio; ICD, implantable cardioverter-defibrillator; QoL, quality of life; VT, ventricular tachycardia.

Explanations:

^a^ICD therapies as a possible surrogate measure of clinically relevant VT/FT.

^b^Although studies were conducted within a timespan of 15 years, three of them were conducted during the last 6 years and this outcome could have been modified by modern drug therapies.

^c^In most studies ablation and ICD implantation were done within a narrow time window and it could be difficult to assign a potential complication to a single procedure. In the two studies comparing ablation to antiarrhythmic drugs, serious adverse events were significantly more frequent in the drug treatment arm.

^d^The results were derived from approximately 48% of the pooled patient population of the three studies reporting QoL outcomes.

^e^High risk of attrition bias and high risk of bias in the measurement of the QoL outcomes for all three studies.

## Discussion

This updated review with a meta-analysis of randomized trials comparing early CA for VT to deferred or no ablation in patients with structural heart disease clearly showed that appropriate ICD shocks and therapies, VT storm, and cardiovascular hospitalizations were significantly reduced with early ablation. There was no benefit from early ablation on all-cause and cardiovascular mortality, and quality of life. Reassuringly, adverse events related to the treatment were equally distributed among the intervention and control arms.

Irrespective of our better understanding of the arrhythmogenic substrate in various etiologies, technological advances, and the use of epicardial approach, the mortality outcomes in this meta-analysis, compared to previously published (10, 11), remained mostly unchanged with early CA vs. deferred of no ablation in patients with structural heart disease and an ICD implanted. The cause for such a lack of improvement might be the lack of tools providing complete single-stepped elimination of the substrate, but more probably it is the underlying disease process that progresses over time and creates new substrate or leads to pump failure and death. It could be equally possible that modern heart failure drug therapies and CRT devices actually improve survival even in patients with VT and no ablation or deferred ablation. Also, three of the trials included death as a secondary outcome and thus may have been statistically underpowered to detect small but significant differences. Nevertheless, early ablation should not be regarded as a tool to improve survival, unless new data emerge in future clinical trials.

Moreover, the ablation of non-ischemic cardiomyopathy and the use of epicardial approach in two of the included trials did not increase mortality or procedure-related complications, which is reassuring despite the small number of patients. Also, the advantages of early ablation on a significant reduction of ICD therapies, shocks, and VT storm, known from previous publications (10, 11), were preserved. In contrast to a previously published meta-analysis (10), we found a significant reduction in cardiovascular hospitalizations. All these findings may translate into reduced health-related expenditures thus potentially further reducing the burden on the healthcare systems (34).

Overall, the results of the addition of three contemporary trials with more than 300 patients in this updated review reinforce our current understanding that CA for recurrent scar-related VT should be implemented early (35, 36). Interestingly, one of those very recent trials–SURVIVE-VT (31) compared ablation to antiarrhythmic drug treatment similar to an earlier trial–VANISH (27), and in both the adverse events were more frequent in the drug treatment control arm. This further supports the notion of early CA. We also need to note that 100% of the patients in VANISH and PARTITA (*n* = 306 or 28.4% of the pooled patient population) and an unknown number of patients in SMASH-VT and SURVIVE-VT had received their ICD well before the ablation and the adverse events directly related to the device implantation are missed in our meta-analysis. Consequently, we could speculate that adverse events in the control arm might be even more than detected in our review, although we do not believe that the OR would be changed significantly in favor of the ablation. The inclusion of the most recent trials into the current meta-analysis might also explain the observed differences in cardiovascular hospitalization in comparison to the previously published meta-analysis. The new studies implemented newer approaches to ablation resulting in possibly better outcomes, thus ensuring less need for cardiovascular hospitalization.

To the best of our knowledge, this is the first meta-analysis including quality of life after ablation as an outcome. ICD shocks are recognized as a major cause of impaired quality of life and may translate into anxiety and depression, loss of work capacity, and sick leaves. Thus, they represent a serious problem for patients with ICD and their relatives (7). According to the results of the current meta-analysis, quality of life was not improved by early ablation which may seem counter-intuitive. This might be explained by the fact that two out of the five trials assessing this outcome reported their results in a way that was not suitable for inclusion in this meta-analysis (26, 33). In addition, only a fraction of the patients in the other three trials had a complete assessment. Because both VTACH and VANISH did not report significant changes, it seems unlikely that the results of the meta-analysis on quality of life would have been different if these trials had been included. Future studies should implement rigorous data collection on quality of life as an outcome, especially bearing in mind that hard clinical outcomes did not improve after many years of technological advances. In this regard, we need to mention that several trials on ablation for VT in structural heart disease are ongoing or are active but not yet recruiting patients. CAAD-VT (ACTRN12620000045910)^1^, Code STORM (See text footnote 1, ACTRN12620001176954), and EPI-VT (NCT04512911)^2^ will not assess the quality of life. However, this outcome is among the secondary ones in three other trials: MANTRA-VT (See text footnote 2, NCT02303639, unknown recruitment status), PREVENTIVE-VT (See text footnote 2, NCT03421834), and VANISH2 (See text footnote 2, NCT02830360). The latter two trials will evaluate also ICD shock-related anxiety and depression.

The additional quantitative synthesis stratified by LVEF showed that ICD therapies were reduced only in patients with higher LVEF, while the ICD shocks and cardiovascular hospitalizations were reduced only in patients with lower LVEF. However, the trends to benefit in the groups with non-significant change were strong, hence, we do not feel that early ablation should be applied selectively according to the baseline LVEF. Mortality outcomes were not impacted by this stratification; hence, one can speculate that VT recurrence (as reflected by appropriate ICD therapy and shocks) is only a marker for mortality risk. Moreover, there is a known association between ICD shocks and mortality (3–6), but the reduction of shocks in this and previous meta-analyses (10, 11) did not translate into mortality benefit.

### Limitations of evidence

Some differences between the studies included in this meta-analysis could not be accounted for by the statistical measurement of heterogeneity. There were different enrolment criteria in terms of primary/secondary prevention and hemodynamic instability of the qualifying arrhythmia. The differences in the proportion of patients who were on baseline amiodarone therapy and the diverse protocols regarding antiarrhythmic drugs use before and after ablation might have led to some bias in the results. In addition, there was variation in the VT mapping approach as well as ablation end-point definition among the studies. Cross-over of patients who were randomized to the control arm but went on to undergo ablation during follow-up may also have introduced some heterogeneity.

Assessment of outcomes based on etiology and access to the heart was not possible in a study-level meta-analysis. Subgroup analysis based on LVEF used mean/median LVEF reported in the individual studies, hence, the data likely are overlapping and heterogenous and although we purposefully used a random effect model, it is not clear whether this was sufficient to overcome this heterogeneity.

### Limitations of review processes

Assessment of risk of bias and certainty of evidence has an unavoidable element of subjectivity. However, we tried to overcome this by including all authors in the assessment and by discussing all disputed issues until reaching a uniform agreement.

### Implications

The findings of our systematic review and meta-analysis of randomized studies reinforce the use of early ablation in patients with structural heart disease and recurrent VT with the purpose of reducing ICD therapies and shocks, VT storm, and hospitalizations for cardiovascular reasons, without increasing the complications related to the treatment. Unless new convincing data emerge, ablation should not be used with the purpose of reducing mortality. Future studies with rigorous data collection should shed light on the impact of ablation on health-related quality of life.

## Other information

### Registration and protocol

This systematic review with meta-analysis was registered with INPLASY on June 19, 2022. The protocol was amended on June 26, 2022 (quality of life added as a secondary outcome and other minor changes done). Both the original and amended versions are available at doi: 10.37766/inplasy2022.6.0080. At the time of the amendment, the search and identification of studies were closed, and the screening for eligibility had started, but the extraction of data had not been started.

## Data availability statement

The original contributions presented in this study are included in the article/Supplementary material, further inquiries can be directed to the corresponding author.

## Author contributions

TS conceived the idea, designed and wrote the protocol, took part in the study search and selection, and data extraction, and wrote the main manuscript draft. MS took part in the study search and selection and data verification. VT took part in the study selection and data extraction. MS and VT wrote parts of the manuscript. All authors independently did risk of bias assessment, participated in the certainty assessment, and critically revised the manuscript and approved it.

## Footnotes

1
https://anzctr.org.au/

2
https://clinicaltrials.gov/
