# Supplementary figures and images for: Outcomes of early catheter ablation for ventricular tachycardia in adult patients with structural heart disease and implantable cardioverter-defibrillator: An updated systematic review and meta-analysis of randomized trials
Source: Front Cardiovasc Med. 2022 Nov 30;9:1063147. doi: 10.3389/fcvm.2022.1063147 (PMC9748081; doi:10.3389/fcvm.2022.1063147)

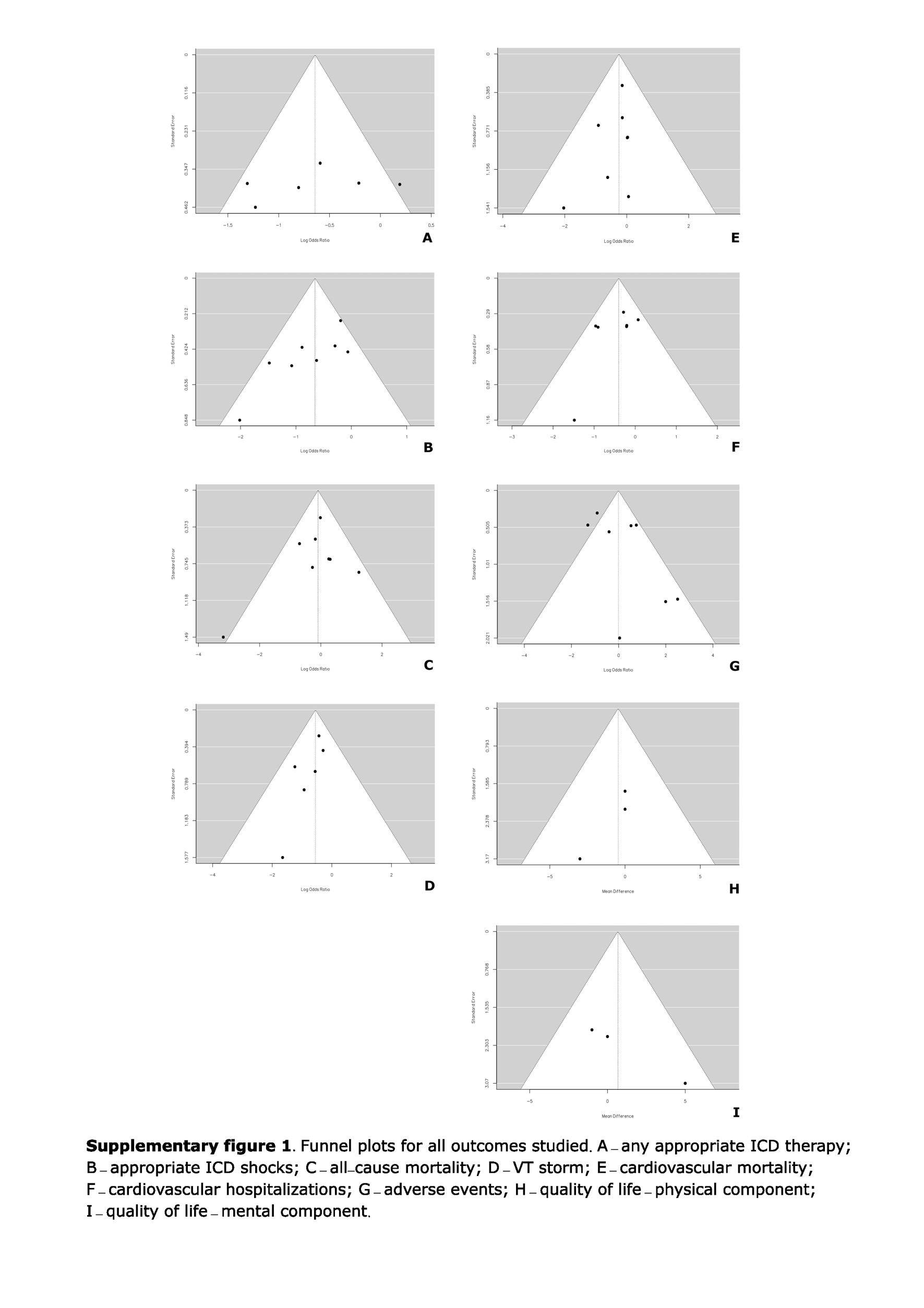

Supplement: Supplementary file 2 [file Image_1.jpg]
